# Dynamics of the COVID-19 epidemic in the post-vaccination period in Korea: a rapid assessment

**DOI:** 10.4178/epih.e2021040

**Published:** 2021-05-27

**Authors:** Kyung-Duk Min, Sangwoo Tak

**Affiliations:** Institute of Health and Environment, Graduate School of Public Health, Seoul National University, Seoul, Korea

**Keywords:** COVID-19, Vaccines, Transmission, Korea

## Abstract

**OBJECTIVES:**

Coronavirus disease 2019 (COVID-19) has had a tremendous impact on public health and socioeconomic conditions globally. Although non-pharmaceutical interventions (NPIs) such as social distancing effectively reduced the incidence of COVID-19, especially in Korea, demand for vaccination has increased to minimize the social costs of NPIs. This study estimated the potential benefits of COVID-19 vaccination in Korea.

**METHODS:**

A mathematical model with vaccinated–susceptible–latent–infectious–recovered compartments was used to simulate the COVID-19 epidemic. The compartments were stratified into age groups of 0-19 years, 20-59 years, and 60 years or older. Based on the Korea Disease Control and Prevention Agency national vaccination plan for the second quarter of 2021, announced on March 15, we developed vaccination scenarios (with 150,000 dose/d and 100% compliance as the main scenario). Comparing scenarios without vaccination or with higher/lower vaccination rates and compliance, we estimated the numbers of COVID-19 cases that will be prevented by vaccination.

**RESULTS:**

The results projected 203,135 cases within a year after April 2021 without vaccination, which would be reduced to 71,248 (64.9% decrease) by vaccination. Supposing a vaccination rate of 150,000 dose/d and 100% compliance, social distancing interventions for those aged 20 or more can be retracted after January 1, 2022.

**CONCLUSIONS:**

We expect COVID-19 vaccination to be effective in Korea. Health authorities should minimize delays in vaccination and vaccine avoidance to maximize the effectiveness of vaccination and end social distancing early.

## INTRODUCTION

Coronavirus disease 2019 (COVID-19) has had a tremendous public health impact globally. As of January 31, 2021, approximately 0.1 billion cases have been reported worldwide, including 2 million deaths [1]. The global gross domestic product is estimated to have decreased by 2% due to the impact of the pandemic [2]. Successful non-pharmaceutical interventions (NPIs), such as social distancing, delaying school opening, and contact tracing combined with self-quarantine, have effectively controlled the epidemic in Korea [3], which had a relatively small number of cases compared to other Organization for Economic Cooperation and Development countries in 2020. However, the number of cases rose significantly starting in November 2020. This surge led to significant shortages in medical staff and hospital beds, affecting the treatment of COVID-19 patients. Although the third wave has been effectively controlled by strict social distancing, including a nationwide ban on gatherings of 5 or more people in cafés or restaurants, social demand for vaccines is increasing to minimize the social costs that arise from social distancing.

Fortunately, the development of COVID-19 vaccines was unprecedentedly rapid, and vaccination started in high-income countries such as the United States and United Kingdom in December 2020 [4]. Health authorities in Korea developed a plan to introduce various COVID-19 vaccines beginning in February 2021, expecting to attain herd immunity by November 2021 [5]. Considering the amount of vaccines and the well-developed infrastructure for their rapid distribution in Korea, vaccination should significantly reduce the COVID-19 burden. However, several questions remain: How much will vaccination reduce the burden of the COVID-19 epidemic? What negative effects might distribution delays or low compliance rates have on the benefits of vaccination? Can vaccination end social distancing interventions? Using mathematical modeling, we examined these questions.

## MATERIALS AND METHODS

A discrete-time compartment model categorizing the entire population into several groups was used. The epidemic dynamics were simulated using parameters such as the number of contacts by an infectious individual, the transmission probability per contact, and the infectious period, starting with the numbers of susceptible, infectious, and recovered people. We used a vaccinated– susceptible–latent–infectious–recovered (V-SLIR) model to represent people who are vaccinated or in the latent period. Since vaccination is prioritized by age, we used a meta-population model that stratified each compartment into 3 subgroups by age (0-19, 20-59, and 60 years or older).

Table 1 describes the parameters incorporated in the model. The latent period was assumed to be 3.5 days, considering an average incubation period of 5.5 days [6] and the fact that infectiousness can develop 2 days before symptoms appear [7]. The infectious period was set as 6.8 days, as the average time between symptom onset and reporting is 4.8 days [8]. The effective contact rate is equal to the product of the transmission probability per contact and contact rate, indicating the number of individuals who can be affected by an infectious individual per day. Considering that the effective contact rate can vary over time, we assumed different effective contact rates for each week for each age group. It was also assumed that transmission between age groups is proportional to the effective contact rate of the infectious age group. For example, the effective contact rates from the child group (aged 0-19) to the middle age group (aged 20-59) is equal to the product of the effective contact rate within the child group (β_c_) and a multiplier for the effective contact rate from the child group to the middle age group (φ_cm_). Although we employed time-varying parameters for the within-group parameters, the between-group parameters were assumed to be constant to reduce the number of parameters. Details of the model, including equations and a schematic diagram, are described in the Supplementary Material 1. Since the effective contact rate is not directly observable, the parameters were calibrated by the daily number of confirmed cases from April 3, 2020 to March 31, 2021 (the data fitting period), using a local random search algorithm [9]. In 2020, a 5-tier social distancing system (levels 1, 1.5, 2, 2.5, and 3) was introduced in Korea. Higher levels prohibited social gatherings more strictly to reduce contact rates. The average effective contact rate in each period categorized by the social distancing level was calculated based on calibrated estimates. For vaccination, we assumed that 5 different vaccine products would be introduced to Korea on April 1, May 15, and August 15. Assumptions regarding the effectiveness of the vaccines were obtained from published papers [10-13].

In designing the vaccine-dispensing scenarios, we prioritized vaccine targets as follows: first dose to those aged ≥ 60, second dose to those aged ≥ 60, first dose to those aged 20-59, and second dose to those aged 20-59 (no vaccination was assumed for those aged 0-19). Cross-vaccination was ruled out and the time gaps between the first and second doses were assumed to be 12 weeks for the AstraZeneca vaccine, 28 days for the Moderna vaccine, and 21 days for the Pfizer vaccine. For simplification, we assumed that the effectiveness of the vaccines from the COVAX facility would be the same as the AstraZeneca vaccine.

Using the V-SLIR model, we predicted COVID-19 epidemics within 1 year after April 2021 (from April 1, 2021 to March 31, 2022) that differed by vaccine-dispensing scenarios developed using some selected vaccination distribution rates, vaccination compliance (coverage), and durations of social distancing interventions. Nine scenarios were constructed as follows: (1) absence of a vaccination program, (2) a vaccination rate of 300,000 dose/d with 100% compliance, (3) a vaccination rate of 250,000 dose/d with 100% compliance, (4) a vaccination rate of 200,000 dose/d with 100% compliance, (5) a vaccination rate of 150,000 dose/d with 100% compliance, (6) a vaccination rate of 100,000 dose/d with 100% compliance, (7) a vaccination rate of 50,000 dose/d with 100% compliance, (8) a vaccination rate of 150,000 dose/d with 90% compliance, and (9) a vaccination rate of 150,000 dose/d with 80% compliance. As the main vaccination scenario, we assumed a distribution rate of 150,000 dose/d and 100% compliance according to the national vaccination plan for the second quarter of 2021. The highest vaccination rate (300,000 dose/d) was based on the vaccine plan for January 28 [5], starting vaccination in Korea in February and attaining herd immunity by November. In the scenario analysis, we assumed that social distancing interventions would be based on the number of confirmed cases, according to how it was implemented in 2020. For example, if the number of confirmed cases increases, the level of social distancing interventions also increases. By comparing the predicted incidence of COVID-19 in each scenario, the potential benefits of the vaccination program along with the effects of the vaccination rate and compliance were presented.

### Ethics statement

This study used aggregated data which is publicly open. No ethical approval was required.

## RESULTS

The mathematical model with a V-SLIR structure replicated the COVID-19 epidemic curve up to March 2021 reasonably well (Supplementary Material 2). The estimated effective contact rate showed that social distancing strategies effectively reduced the effective contact rates in Korea (Supplementary Material 3). Based on the estimated effective contact rate by time period, we assumed the effective contact rates for our analysis of scenarios, as shown in Table 2. Although a 5-tier social distancing system was used in practice, we recategorized the social distancing levels into 4 grades for simplification, supposing the implementation of social distancing interventions based on the epidemic burden of the previous week (i.e., if the number of cases increased, the social distancing grade would increase).

**Figure 1.**
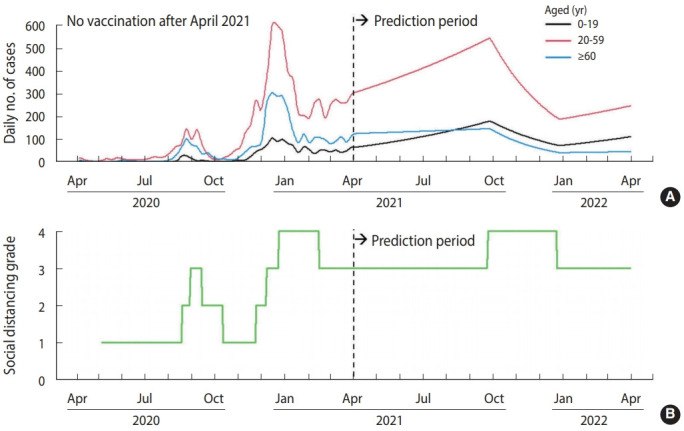
Scenario result assuming absence of a vaccination program from April 2021. (A) Simulated daily number of cases, (B) simulated social distancing grade.

**Figure 2.**
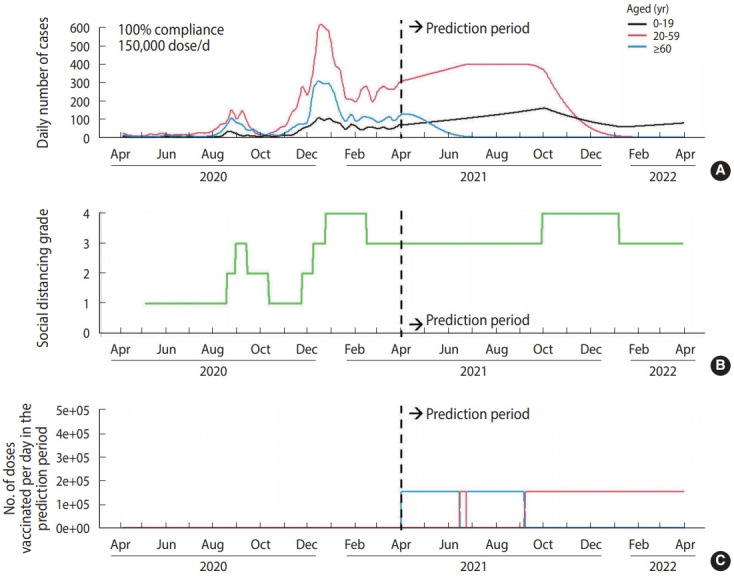
Scenario result (A) simulated daily number of cases, (B) simulated social distancing grade, and (C) simulated number of vaccinations per day assuming a vaccination distribution rate of 150,000 dose/d and 100% compliance.

**Figure 3.**
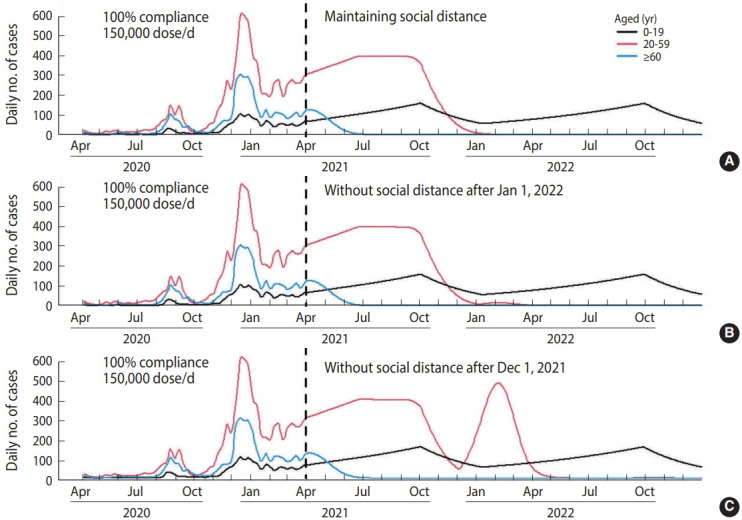
Scenario results assuming a vaccination distribution rate of 150,000 dose/d and 100% compliance (A) with social distancing, (B) without social distancing of those aged 20 or more after January 2022, and (C) without social distancing of those aged 20 or more after December 2021.

**Figure 4.**
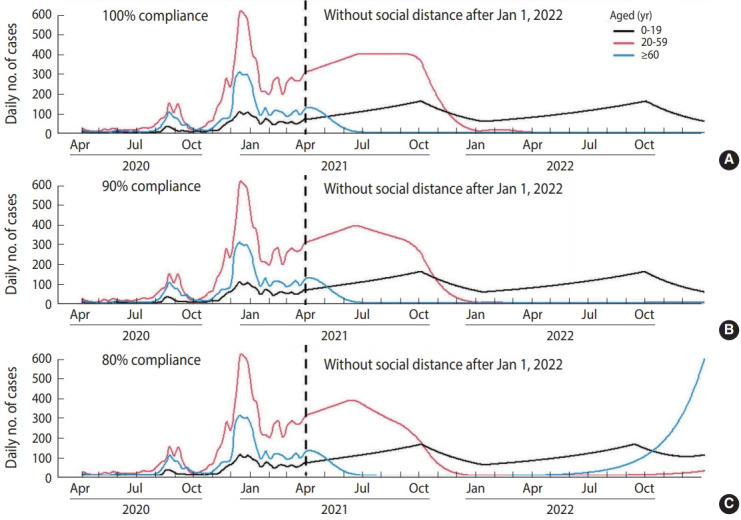
Scenario results assuming a vaccination distribution rate of 150,000 dose/d and (A) 100%, (B) 90%, or (C) 80% compliance, without social distancing of those aged 20 or more after January 2022.

Supposing no vaccination after April 2021 (Figure 1), there would be 203,135 cumulative cases within 1 year after April 2021. With a vaccination distribution rate of 150,000 dose/d and 100% compliance (Figure 2), the cumulative number of cases was projected to decrease to 71,248. However, the number of cases increased if we assumed a lower vaccination rate (i.e., delayed vaccine distribution) (Table 3). In case of lower compliance, the number of cases in those aged ≥ 60 would increase, but the number of cases in those aged 20-59 would decrease. Supposing a vaccination rate of 150,000 dose/d and 100% compliance, social distancing interventions for those aged 20 or more can be lifted after January 1, 2022 (Figure 3). However, in the case of 80% compliance, the end of social distancing after January 1, 2022 would cause a resurgence of the epidemic in 2022 (Figure 4).

## DISCUSSION

A V-SLIR model incorporating a time-varying effective contact rate was used to predict epidemic dynamics in the post-vaccination period in Korea. This model showed that the social distancing strategy implemented in 2020 decreased the effective contact rate in Korea. In the analysis, we found significant benefits from vaccinations when administered at 150,000 dose/d with 100% compliance. However, if vaccine distribution is delayed (a lower vaccination rate), the benefits would be reduced. Even with the vaccination scenario, social distancing among those aged 20-59 and aged ≥ 60 (target population for vaccination) should be maintained until herd immunity is achieved.

The benefits of vaccination in reducing the incidence of COVID-19 will help in the decision as to when social distancing for adults can be lifted. We believe that, with no delays in vaccination distribution, social distancing interventions could be lifted as early as January 1, 2022 without a resurgence of the epidemic in 2022. In this study, we found that lower compliance rates led to fewer cases in the prediction period. This unexpected finding was attributed to early vaccination of those aged 20-59 due to fewer vaccinations in the ≥ 60 group. The reason for this is that the number of the target population for the ≥ 60 group decreased in the scenario of lower compliance, which in turn would cause early completion of vaccination in the old age group and early introduction of vaccination in the middle age group. Although this might reduce the total number of cases, it would increase the number of cases in the ≥ 60 group, possibly causing more deaths considering the higher case-fatality rate in this age group.

The results of our model presented specific numbers regarding the effects of vaccination on the number of confirmed cases and the end of social distancing. However, these figures could be easily affected by various model assumptions. Therefore, the following study limitations should be considered when interpreting our results. First, the parameters we use may need to be adjusted as necessary. For example, variants of the SARS-CoV-2 virus could increase the effective contact rate depending on their behavior [14]. In addition, the duration of immunity from a vaccine or natural infection could be finite, although we assumed at least 1-year-long immunity to simplify the models. Second, the Korea Disease Control and Prevention Agency prioritized vaccination for those aged 65 or more, followed by those aged 18-64 years [5]. However, we categorized the age groups as 0-19, 20-59, and ≥ 60, so our stratification does not fully represent the official vaccination prioritization. Third, our compartment models cannot incorporate individual characteristics, such as occupation and comorbidities, which affect susceptibility to the infection and transmissibility. Fourth, lower compliance resulted in lower vaccine coverage in this study, but in practice, lower compliance could also result in a slower pace of vaccination. Fifth, the between-group effective contact rates could have been underestimated. In the model, we employed time-varying parameters for within-group effective contact rates for each week, but constant multiplier parameters for between-group effective contact rates in order to reduce the number of parameters. Although we estimated the effective contact rates between groups as being close to 0, those could have been underestimations due to the restricted assumptions for the between-group parameters, instead of reflecting the true transmission dynamics between groups. Finally, adverse effects of vaccination, such as anaphylaxis, were not included in the model; hence the duration and required medical resources may have been underestimated. The effect of a vaccination campaign might improve with measures to prepare for potential adverse outcomes.

In conclusion, vaccination according to the national plan could substantially reduce the number of confirmed cases under social distancing interventions. Health authorities should maximize compliance and the pace of vaccination for a successful vaccination campaign.

## Figures and Tables

**Table 1.** Parameters used in the models

Category	Parameter	Value
Transmission related	Latent period	3.5 d^1^
	Infectious period	6.8 d^2^
	Effective contact rate^3^	Calibrated
Vaccine related^4^	Maximum daily no. of vaccinations	150,000 dose/d^5^
	AstraZeneca	
	Introduction date	April 1
	Total no. of doses	20 million
	Effectiveness (first dose)	0.7
	Effectiveness (second dose)	0.7
	Interval between doses	12 wk
	Moderna	
	Introduction date	May 15
	Total no. of doses	40 million
	Effectiveness (first dose)	0.94
	Effectiveness (second dose)	0.94
	Interval between doses	28 d
	Janssen	
	Introduction date	May 15
	Total no. of doses	6 million
	Effectiveness (first dose)	0.66
	Pfizer	
	Introduction date	August 15
	Total no. of doses	20 million
	Effectiveness (first dose)	0.52
	Effectiveness (second dose)	0.95
	Interval between doses	21 d
	COVAX facility	Same as AstraZeneca

1 Incubation period 5.5 days; transmissible 2 days before symptoms appear.

2 Infectious period (from symptom onset to isolation) 4.8 days; transmissible 2 days before symptoms appear.

3 Time-dependent parameter (weekly).

4 Introduction date of each vaccine based on the January 28 vaccine plan; the effectiveness was based on previous publications.

5 Assumption based on the vaccine plan in Korea starting in mid-February and ending for the entire population in November.

**Table 2.** Assumed effective contact rate in the scenario supposing adjustment of social distancing interventions based on the epidemic burden^1^

Social distancing grade	Assumed estimated effective contact rate	Criteria for changing the social distancing grade	
		Change	Criteria^2^
Grade 1 (weakest)	0.243	From 2 to 1	66.1
Grade 2	0.206	From 1 to 2	280.9
		From 3 to 2	125.0
Grade 3	0.154	From 2 to 3	476.7
		From 4 to 3	333.1
Grade 4 (strongest)	0.132	From 3 to 4	856.7
Grade 0 (no social distancing)^3^	0.441	-	

1 In our scenario analysis, we supposed that the social distancing grade will be adjusted based on the number of confirmed cases; For example, if the number of confirmed cases increased to over 856.7, the social distancing grade would increase from 3 to 4, consequently decreasing the effective contact rate (from 0.154 to 0.132); The assumed effective contact rate according to the social distancing grade and criteria for changing social distancing grade were based on the estimated effective contact rate in model calibration; Although the current social distancing policy is more complicated, we used only four grades of social distancing stringency for simplification.

2 Mean number of daily confirmed cases between the day of increasing/decreasing the social distancing grade and 1 week before the day.

3 As the basic reproduction number and infectious period were assumed to be 3.0 and 6.8, respectively, the effective contact rate without any social distancing measures was assumed to be 0.441 (=3.0/6.8).

**Table 3.** Results of the analysis of various scenarios^1^

No.	Scenario description		Cumulative no. of confirmed cases^2^			
	Vaccination rate (dose/d)	Compliance, %	Age (yr)			
			0-19	20-59	≥60	Total
1	-	-	40,647	125,848	36,640	203,135
2	300,000	100	35,338	31,758	4,152	71,248
3	250,000	100	35,331	39,026	4,485	78,842
4	200,000	100	35,372	52,100	4,953	92,425
5	150,000	100	35,351	80,890	5,659	121,900
6	100,000	100	35,199	110,040	6,878	152,117
7	50,000	100	35,164	118,345	9,720	163,229
8	150,000	90	35,354	70,546	5,678	111,578
9	150,000	80	35,364	62,013	5,728	103,105

1 1n this study, we analyzed 9 scenarios to predict the epidemic of coronavirus disease 2019 (COVID-19) in Korea in 2021; Te first scenario represented an epidemic situation without a vaccination program; The other 8 scenarios indicated the epidemics with vaccination programs with different vaccination rates and compliance levels; Each scenario analysis generated the cumulative number of cases in 2021 (from January 1, 2021 to December 31, 2021) for each age group and the number of days at various social distancing grades (lower grades represent less stringent interventions).

2 Projected number of confirmed cases from April 1, 2021 to March 31, 2022.

## SUPPLEMENTARY MATERIALS

Supplementary Material 1. Equations and schematic diagram for mathematical model in this study

Supplementary Material 2. Model calibration results and estimated effective contact rate for each age group.

Supplementary Material 3. Effective contact rate for each age group and each period estimated by calibration
