# Supplementary material for: Dynamics of the COVID-19 epidemic in the post-vaccination period in Korea: a rapid assessment
Source: Epidemiol Health. 2021 May 27;43:e2021040. doi: 10.4178/epih.e2021040 (PMC8289478; doi:10.4178/epih.e2021040)
Supplement: Supplementary Material 1. — Equations and schematic diagram for mathematical model in this study [file epih-43-e2021040-suppl1.docx]

**Supplementary Material 1. Equations and schematic diagram for mathematical model in this study**

Schematic diagram and equations incorporated in the V-SLIR model with three age groups are shown below:

| **Schematic diagram** |
| --- |
| 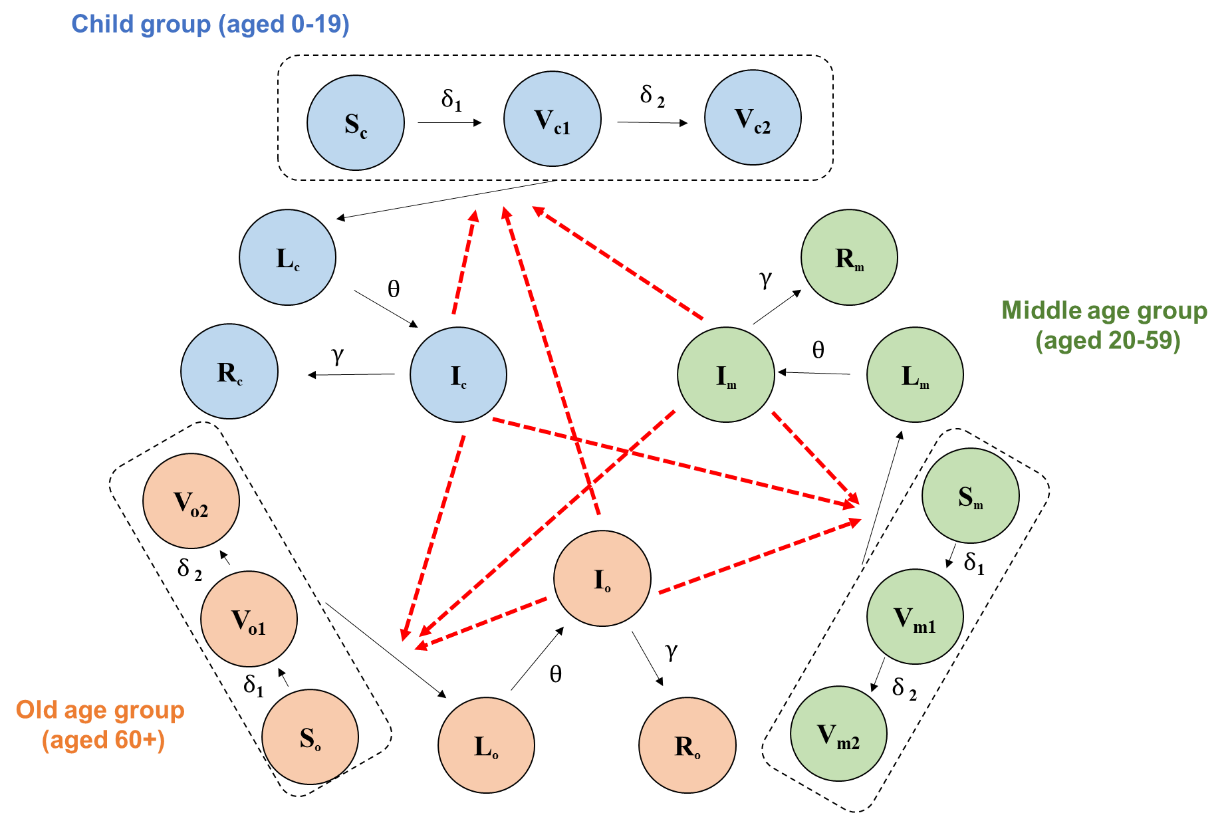 |
| **Equations for the group aged 0-19** |
| $\frac{\boldsymbol{d}\boldsymbol{V}_{\boldsymbol{1}\boldsymbol{c}}}{\boldsymbol{dt}}\boldsymbol{=}\boldsymbol{\delta}_{\boldsymbol{1}}\boldsymbol{S}_{\boldsymbol{c}}\boldsymbol{-}{\left( \boldsymbol{1-}\boldsymbol{\varepsilon}_{\boldsymbol{1}} \right)\left( \boldsymbol{V}_{\boldsymbol{1}\boldsymbol{c}}\boldsymbol{/}\boldsymbol{N}_{\boldsymbol{c}} \right)\boldsymbol{\{\beta(t)}}_{\boldsymbol{c}}\boldsymbol{I}_{\boldsymbol{c}}\boldsymbol{-}\boldsymbol{\varphi}_{\boldsymbol{m}\boldsymbol{c}}\boldsymbol{\beta(t)}_{\boldsymbol{m}}\boldsymbol{I}_{\boldsymbol{m}}\boldsymbol{-}\boldsymbol{\varphi}_{\boldsymbol{o}\boldsymbol{c}}\boldsymbol{\beta(t)}_{\boldsymbol{o}}\boldsymbol{I}_{\boldsymbol{o}}\boldsymbol{\}-}\boldsymbol{\delta}_{\boldsymbol{2}}\boldsymbol{V}_{\boldsymbol{1}\boldsymbol{c}}$  $\frac{\boldsymbol{d}\boldsymbol{V}_{\boldsymbol{2}\boldsymbol{c}}}{\boldsymbol{dt}}\boldsymbol{=}\boldsymbol{\delta}_{\boldsymbol{2}}\boldsymbol{V}_{\boldsymbol{1}\boldsymbol{c}}\boldsymbol{-}{\left( \boldsymbol{1-}\boldsymbol{\varepsilon}_{\boldsymbol{2}} \right)\left( \boldsymbol{V}_{\boldsymbol{2}\boldsymbol{c}}\boldsymbol{/}\boldsymbol{N}_{\boldsymbol{c}} \right)\boldsymbol{\{\beta(t)}}_{\boldsymbol{c}}\boldsymbol{I}_{\boldsymbol{c}}\boldsymbol{-}\boldsymbol{\varphi}_{\boldsymbol{m}\boldsymbol{c}}\boldsymbol{\beta(t)}_{\boldsymbol{m}}\boldsymbol{I}_{\boldsymbol{m}}\boldsymbol{-}\boldsymbol{\varphi}_{\boldsymbol{o}\boldsymbol{c}}\boldsymbol{\beta(t)}_{\boldsymbol{o}}\boldsymbol{I}_{\boldsymbol{o}}\boldsymbol{\}}$  $\frac{\boldsymbol{d}\boldsymbol{S}_{\boldsymbol{c}}}{\boldsymbol{dt}}\boldsymbol{=-}\boldsymbol{\beta(t)}_{\boldsymbol{c}}\boldsymbol{I}_{\boldsymbol{c}}\left( \boldsymbol{S}_{\boldsymbol{c}}\boldsymbol{/}\boldsymbol{N}_{\boldsymbol{c}} \right)\boldsymbol{-}\boldsymbol{\varphi}_{\boldsymbol{m}\boldsymbol{c}}\boldsymbol{\beta(t)}_{\boldsymbol{m}}\boldsymbol{I}_{\boldsymbol{m}}\left( \boldsymbol{S}_{\boldsymbol{c}}\boldsymbol{/}\boldsymbol{N}_{\boldsymbol{c}} \right)\boldsymbol{-}\boldsymbol{\varphi}_{\boldsymbol{o}\boldsymbol{c}}\boldsymbol{\beta(t)}_{\boldsymbol{o}}\boldsymbol{I}_{\boldsymbol{o}}\left( \boldsymbol{S}_{\boldsymbol{c}}\boldsymbol{/}\boldsymbol{N}_{\boldsymbol{c}} \right)\boldsymbol{-}\boldsymbol{\delta}_{\boldsymbol{1}}\boldsymbol{S}_{\boldsymbol{c}}$  $\frac{\boldsymbol{d}\boldsymbol{L}_{\boldsymbol{c}}}{\boldsymbol{dt}}\boldsymbol{=}\boldsymbol{\beta(t)}_{\boldsymbol{c}}\boldsymbol{I}_{\boldsymbol{c}}\left( \boldsymbol{S}_{\boldsymbol{c}}\boldsymbol{/}\boldsymbol{N}_{\boldsymbol{c}} \right) \boldsymbol{+} \boldsymbol{\varphi}_{\boldsymbol{m}\boldsymbol{c}}\boldsymbol{\beta(t)}_{\boldsymbol{m}}\boldsymbol{I}_{\boldsymbol{m}}\left( \boldsymbol{S}_{\boldsymbol{c}}\boldsymbol{/}\boldsymbol{N}_{\boldsymbol{c}} \right)\boldsymbol{+}\boldsymbol{\varphi}_{\boldsymbol{o}\boldsymbol{c}}\boldsymbol{\beta(t)}_{\boldsymbol{o}}\boldsymbol{I}_{\boldsymbol{o}}\left( \boldsymbol{S}_{\boldsymbol{c}}\boldsymbol{/}\boldsymbol{N}_{\boldsymbol{c}} \right)\boldsymbol{+}{\left( \boldsymbol{1-}\boldsymbol{\varepsilon}_{\boldsymbol{1}} \right)\left( \boldsymbol{V}_{\boldsymbol{1}\boldsymbol{c}}\boldsymbol{/}\boldsymbol{N}_{\boldsymbol{c}} \right)\boldsymbol{\{\beta(t)}}_{\boldsymbol{c}}\boldsymbol{I}_{\boldsymbol{c}}\boldsymbol{-}\boldsymbol{\varphi}_{\boldsymbol{m}\boldsymbol{c}}\boldsymbol{\beta(t)}_{\boldsymbol{m}}\boldsymbol{I}_{\boldsymbol{m}}\boldsymbol{-}\boldsymbol{\varphi}_{\boldsymbol{o}\boldsymbol{c}}\boldsymbol{\beta(t)}_{\boldsymbol{o}}\boldsymbol{I}_{\boldsymbol{o}}\boldsymbol{\}+}{\left( \boldsymbol{1-}\boldsymbol{\varepsilon}_{\boldsymbol{2}} \right)\left( \boldsymbol{V}_{\boldsymbol{2}\boldsymbol{c}}\boldsymbol{/}\boldsymbol{N}_{\boldsymbol{c}} \right)\boldsymbol{\{\beta(t)}}_{\boldsymbol{c}}\boldsymbol{I}_{\boldsymbol{c}}\boldsymbol{-}\boldsymbol{\varphi}_{\boldsymbol{m}\boldsymbol{c}}\boldsymbol{\beta(t)}_{\boldsymbol{m}}\boldsymbol{I}_{\boldsymbol{m}}\boldsymbol{-}\boldsymbol{\varphi}_{\boldsymbol{o}\boldsymbol{c}}\boldsymbol{\beta(t)}_{\boldsymbol{o}}\boldsymbol{I}_{\boldsymbol{o}}\boldsymbol{\} -\theta}\boldsymbol{L}_{\boldsymbol{c}}$  $\frac{\boldsymbol{d}\boldsymbol{I}_{\boldsymbol{c}}}{\boldsymbol{dt}}\boldsymbol{=\theta}\boldsymbol{L}_{\boldsymbol{c}}\boldsymbol{-\gamma}\boldsymbol{I}_{\boldsymbol{c}}$  $\frac{\boldsymbol{d}\boldsymbol{R}_{\boldsymbol{c}}}{\boldsymbol{dt}}\boldsymbol{=\gamma}\boldsymbol{I}_{\boldsymbol{c}}$ |
| **Equations for the group aged 20-59** |
| $\frac{\boldsymbol{d}\boldsymbol{V}_{\boldsymbol{1}\boldsymbol{m}}}{\boldsymbol{dt}}\boldsymbol{=}\boldsymbol{\delta}_{\boldsymbol{1}}\boldsymbol{S}_{\boldsymbol{m}}\boldsymbol{-}{\left( \boldsymbol{1-}\boldsymbol{\varepsilon}_{\boldsymbol{1}} \right)\left( \boldsymbol{V}_{\boldsymbol{1}\boldsymbol{m}}\boldsymbol{/}\boldsymbol{N}_{\boldsymbol{m}} \right)\boldsymbol{\{\beta(t)}}_{\boldsymbol{m}}\boldsymbol{I}_{\boldsymbol{m}}\boldsymbol{-}\boldsymbol{\varphi}_{\boldsymbol{c}\boldsymbol{m}}\boldsymbol{\beta(t)}_{\boldsymbol{c}}\boldsymbol{I}_{\boldsymbol{c}}\boldsymbol{-}\boldsymbol{\varphi}_{\boldsymbol{om}}\boldsymbol{\beta(t)}_{\boldsymbol{o}}\boldsymbol{I}_{\boldsymbol{o}}\boldsymbol{\}-}\boldsymbol{\delta}_{\boldsymbol{2}}\boldsymbol{V}_{\boldsymbol{1}\boldsymbol{m}}$  $\frac{\boldsymbol{d}\boldsymbol{V}_{\boldsymbol{2}\boldsymbol{m}}}{\boldsymbol{dt}}\boldsymbol{=}\boldsymbol{\delta}_{\boldsymbol{2}}\boldsymbol{V}_{\boldsymbol{1}\boldsymbol{m}}\boldsymbol{-}{\left( \boldsymbol{1-}\boldsymbol{\varepsilon}_{\boldsymbol{2}} \right)\left( \boldsymbol{V}_{\boldsymbol{2}\boldsymbol{m}}\boldsymbol{/}\boldsymbol{N}_{\boldsymbol{m}} \right)\boldsymbol{\{\beta(t)}}_{\boldsymbol{m}}\boldsymbol{I}_{\boldsymbol{m}}\boldsymbol{-}\boldsymbol{\varphi}_{\boldsymbol{c}\boldsymbol{m}}\boldsymbol{\beta(t)}_{\boldsymbol{c}}\boldsymbol{I}_{\boldsymbol{c}}\boldsymbol{-}\boldsymbol{\varphi}_{\boldsymbol{om}}\boldsymbol{\beta(t)}_{\boldsymbol{o}}\boldsymbol{I}_{\boldsymbol{o}}\boldsymbol{\}}$  $\frac{\boldsymbol{d}\boldsymbol{S}_{\boldsymbol{m}}}{\boldsymbol{dt}}\boldsymbol{=-}\boldsymbol{\beta(t)}_{\boldsymbol{m}}\boldsymbol{I}_{\boldsymbol{m}}\left( \boldsymbol{S}_{\boldsymbol{m}}\boldsymbol{/}\boldsymbol{N}_{\boldsymbol{m}} \right)\boldsymbol{-}\boldsymbol{\varphi}_{\boldsymbol{c}\boldsymbol{m}}\boldsymbol{\beta(t)}_{\boldsymbol{c}}\boldsymbol{I}_{\boldsymbol{c}}\left( \boldsymbol{S}_{\boldsymbol{m}}\boldsymbol{/}\boldsymbol{N}_{\boldsymbol{m}} \right)\boldsymbol{-}\boldsymbol{\varphi}_{\boldsymbol{om}}\boldsymbol{\beta(t)}_{\boldsymbol{o}}\boldsymbol{I}_{\boldsymbol{o}}\left( \boldsymbol{S}_{\boldsymbol{m}}\boldsymbol{/}\boldsymbol{N}_{\boldsymbol{m}} \right)\boldsymbol{-}\boldsymbol{\delta}_{\boldsymbol{1}}\boldsymbol{S}_{\boldsymbol{m}}$  $\frac{\boldsymbol{d}\boldsymbol{L}_{\boldsymbol{m}}}{\boldsymbol{dt}}\boldsymbol{=}\boldsymbol{\beta(t)}_{\boldsymbol{m}}\boldsymbol{I}_{\boldsymbol{m}}\left( \boldsymbol{S}_{\boldsymbol{m}}\boldsymbol{/}\boldsymbol{N}_{\boldsymbol{m}} \right)\boldsymbol{+}\boldsymbol{\varphi}_{\boldsymbol{c}\boldsymbol{m}}\boldsymbol{\beta(t)}_{\boldsymbol{c}}\boldsymbol{I}_{\boldsymbol{c}}\left( \boldsymbol{S}_{\boldsymbol{m}}\boldsymbol{/}\boldsymbol{N}_{\boldsymbol{m}} \right)\boldsymbol{+}\boldsymbol{\varphi}_{\boldsymbol{om}}\boldsymbol{\beta(t)}_{\boldsymbol{o}}\boldsymbol{I}_{\boldsymbol{o}}\left( \boldsymbol{S}_{\boldsymbol{m}}\boldsymbol{/}\boldsymbol{N}_{\boldsymbol{m}} \right)\boldsymbol{+}{\left( \boldsymbol{1-}\boldsymbol{\varepsilon}_{\boldsymbol{1}} \right)\left( \boldsymbol{V}_{\boldsymbol{1}\boldsymbol{m}}\boldsymbol{/}\boldsymbol{N}_{\boldsymbol{m}} \right)\boldsymbol{\{\beta(t)}}_{\boldsymbol{m}}\boldsymbol{I}_{\boldsymbol{m}}\boldsymbol{-}\boldsymbol{\varphi}_{\boldsymbol{c}\boldsymbol{m}}\boldsymbol{\beta(t)}_{\boldsymbol{c}}\boldsymbol{I}_{\boldsymbol{c}}\boldsymbol{-}\boldsymbol{\varphi}_{\boldsymbol{om}}\boldsymbol{\beta(t)}_{\boldsymbol{o}}\boldsymbol{I}_{\boldsymbol{o}}\boldsymbol{\}+}{\left( \boldsymbol{1-}\boldsymbol{\varepsilon}_{\boldsymbol{2}} \right)\left( \boldsymbol{V}_{\boldsymbol{2}\boldsymbol{m}}\boldsymbol{/}\boldsymbol{N}_{\boldsymbol{m}} \right)\boldsymbol{\{\beta(t)}}_{\boldsymbol{m}}\boldsymbol{I}_{\boldsymbol{m}}\boldsymbol{-}\boldsymbol{\varphi}_{\boldsymbol{c}\boldsymbol{m}}\boldsymbol{\beta(t)}_{\boldsymbol{c}}\boldsymbol{I}_{\boldsymbol{c}}\boldsymbol{-}\boldsymbol{\varphi}_{\boldsymbol{om}}\boldsymbol{\beta(t)}_{\boldsymbol{o}}\boldsymbol{I}_{\boldsymbol{o}}\boldsymbol{\} -\theta}\boldsymbol{L}_{\boldsymbol{m}}$  $\frac{\boldsymbol{d}\boldsymbol{I}_{\boldsymbol{m}}}{\boldsymbol{dt}}\boldsymbol{=\theta}\boldsymbol{L}_{\boldsymbol{m}}\boldsymbol{-\gamma}\boldsymbol{I}_{\boldsymbol{m}}$  $\frac{\boldsymbol{d}\boldsymbol{R}_{\boldsymbol{m}}}{\boldsymbol{dt}}\boldsymbol{=\gamma}\boldsymbol{I}_{\boldsymbol{m}}$ |
| **Equations for the group aged 60+** |
| $\frac{\boldsymbol{d}\boldsymbol{V}_{\boldsymbol{1}\boldsymbol{o}}}{\boldsymbol{dt}}\boldsymbol{=}\boldsymbol{\delta}_{\boldsymbol{1}}\boldsymbol{S}_{\boldsymbol{o}}\boldsymbol{-}{\left( \boldsymbol{1-}\boldsymbol{\varepsilon}_{\boldsymbol{1}} \right)\left( \boldsymbol{V}_{\boldsymbol{1}\boldsymbol{o}}\boldsymbol{/}\boldsymbol{N}_{\boldsymbol{o}} \right)\boldsymbol{\{\beta(t)}}_{\boldsymbol{o}}\boldsymbol{I}_{\boldsymbol{o}}\boldsymbol{-}\boldsymbol{\varphi}_{\boldsymbol{co}}\boldsymbol{\beta(t)}_{\boldsymbol{c}}\boldsymbol{I}_{\boldsymbol{c}}\boldsymbol{-}\boldsymbol{\varphi}_{\boldsymbol{mo}}\boldsymbol{\beta(t)}_{\boldsymbol{m}}\boldsymbol{I}_{\boldsymbol{m}}\boldsymbol{\}-}\boldsymbol{\delta}_{\boldsymbol{2}}\boldsymbol{V}_{\boldsymbol{1}\boldsymbol{o}}$  $\frac{\boldsymbol{d}\boldsymbol{V}_{\boldsymbol{2}\boldsymbol{o}}}{\boldsymbol{dt}}\boldsymbol{=}\boldsymbol{\delta}_{\boldsymbol{2}}\boldsymbol{V}_{\boldsymbol{1}\boldsymbol{o}}\boldsymbol{-}{\left( \boldsymbol{1-}\boldsymbol{\varepsilon}_{\boldsymbol{2}} \right)\left( \boldsymbol{V}_{\boldsymbol{2}\boldsymbol{o}}\boldsymbol{/}\boldsymbol{N}_{\boldsymbol{o}} \right)\boldsymbol{\{\beta(t)}}_{\boldsymbol{o}}\boldsymbol{I}_{\boldsymbol{o}}\boldsymbol{-}\boldsymbol{\varphi}_{\boldsymbol{c}\boldsymbol{o}}\boldsymbol{\beta(t)}_{\boldsymbol{c}}\boldsymbol{I}_{\boldsymbol{c}}\boldsymbol{-}\boldsymbol{\varphi}_{\boldsymbol{mo}}\boldsymbol{\beta(t)}_{\boldsymbol{m}}\boldsymbol{I}_{\boldsymbol{m}}\boldsymbol{\}}$  $\frac{\boldsymbol{d}\boldsymbol{S}_{\boldsymbol{o}}}{\boldsymbol{dt}}\boldsymbol{=-}\boldsymbol{\beta(t)}_{\boldsymbol{o}}\boldsymbol{I}_{\boldsymbol{o}}\left( \boldsymbol{S}_{\boldsymbol{o}}\boldsymbol{/}\boldsymbol{N}_{\boldsymbol{o}} \right)\boldsymbol{-}\boldsymbol{\varphi}_{\boldsymbol{c}\boldsymbol{o}}\boldsymbol{\beta(t)}_{\boldsymbol{c}}\boldsymbol{I}_{\boldsymbol{c}}\left( \boldsymbol{S}_{\boldsymbol{o}}\boldsymbol{/}\boldsymbol{N}_{\boldsymbol{o}} \right)\boldsymbol{-}\boldsymbol{\varphi}_{\boldsymbol{mo}}\boldsymbol{\beta(t)}_{\boldsymbol{m}}\boldsymbol{I}_{\boldsymbol{m}}\left( \boldsymbol{S}_{\boldsymbol{o}}\boldsymbol{/}\boldsymbol{N}_{\boldsymbol{o}} \right)\boldsymbol{-}\boldsymbol{\delta}_{\boldsymbol{1}}\boldsymbol{S}_{\boldsymbol{o}}$  $\frac{\boldsymbol{d}\boldsymbol{L}_{\boldsymbol{o}}}{\boldsymbol{dt}}\boldsymbol{=}\boldsymbol{\beta(t)}_{\boldsymbol{o}}\boldsymbol{I}_{\boldsymbol{o}}\left( \boldsymbol{S}_{\boldsymbol{o}}\boldsymbol{/}\boldsymbol{N}_{\boldsymbol{o}} \right) \boldsymbol{+} \boldsymbol{\varphi}_{\boldsymbol{c}\boldsymbol{o}}\boldsymbol{\beta(t)}_{\boldsymbol{c}}\boldsymbol{I}_{\boldsymbol{c}}\left( \boldsymbol{S}_{\boldsymbol{o}}\boldsymbol{/}\boldsymbol{N}_{\boldsymbol{o}} \right)\boldsymbol{+}\boldsymbol{\varphi}_{\boldsymbol{mo}}\boldsymbol{\beta(t)}_{\boldsymbol{m}}\boldsymbol{I}_{\boldsymbol{m}}\left( \boldsymbol{S}_{\boldsymbol{o}}\boldsymbol{/}\boldsymbol{N}_{\boldsymbol{o}} \right)\boldsymbol{+}{\left( \boldsymbol{1-}\boldsymbol{\varepsilon}_{\boldsymbol{1}} \right)\left( \boldsymbol{V}_{\boldsymbol{1}\boldsymbol{o}}\boldsymbol{/}\boldsymbol{N}_{\boldsymbol{o}} \right)\boldsymbol{\{\beta(t)}}_{\boldsymbol{o}}\boldsymbol{I}_{\boldsymbol{o}}\boldsymbol{-}\boldsymbol{\varphi}_{\boldsymbol{co}}\boldsymbol{\beta(t)}_{\boldsymbol{c}}\boldsymbol{I}_{\boldsymbol{c}}\boldsymbol{-}\boldsymbol{\varphi}_{\boldsymbol{mo}}\boldsymbol{\beta(t)}_{\boldsymbol{m}}\boldsymbol{I}_{\boldsymbol{m}}\boldsymbol{\}+}{\left( \boldsymbol{1-}\boldsymbol{\varepsilon}_{\boldsymbol{2}} \right)\left( \boldsymbol{V}_{\boldsymbol{2}\boldsymbol{o}}\boldsymbol{/}\boldsymbol{N}_{\boldsymbol{o}} \right)\boldsymbol{\{\beta(t)}}_{\boldsymbol{o}}\boldsymbol{I}_{\boldsymbol{o}}\boldsymbol{-}\boldsymbol{\varphi}_{\boldsymbol{c}\boldsymbol{o}}\boldsymbol{\beta(t)}_{\boldsymbol{c}}\boldsymbol{I}_{\boldsymbol{c}}\boldsymbol{-}\boldsymbol{\varphi}_{\boldsymbol{mo}}\boldsymbol{\beta(t)}_{\boldsymbol{m}}\boldsymbol{I}_{\boldsymbol{m}}\boldsymbol{\} -\theta}\boldsymbol{L}_{\boldsymbol{o}}$  $\frac{\boldsymbol{d}\boldsymbol{I}_{\boldsymbol{o}}}{\boldsymbol{dt}}\boldsymbol{=\theta}\boldsymbol{L}_{\boldsymbol{o}}\boldsymbol{-\gamma}\boldsymbol{I}_{\boldsymbol{o}}$  $\frac{\boldsymbol{d}\boldsymbol{R}_{\boldsymbol{o}}}{\boldsymbol{dt}}\boldsymbol{=\gamma}\boldsymbol{I}_{\boldsymbol{o}}$ |
| *Where,*  *V_1_: vaccinated group (first dose)*  *V_2_: vaccinated group (second dose)*  *S: susceptible population*  *L: population in the latent period*  *I: infectious population*  *R: recovered population (hospitalized and isolated population)*  *N: Population size*  $\beta(t)$*: time varying effective contact rate within age group*  $\theta$*: rate at which a latent individual becomes infectious*  $\gamma$*: detection rate (reciprocal of infectious period*  *φ: multiplier for effective contact rate between age groups*  $\delta_{1}$*: vaccination rate for first doses*  $\delta_{2}$*: vaccination rate for second doses*  *subscript “c” indicates individuals aged 0-19, “m” indicates individuals aged 20-59, and “o” indicates individuals aged 60+*  *subscript “mc” indicates from individuals aged 20-59 to individuals aged 0-19*  *subscript “oc” indicates from individuals aged 60+ to individuals aged 0-19*  *subscript “cm” indicates from individuals aged 0-19 to individuals aged 20-59*  *subscript “om” indicates from individuals aged 60+ to individuals aged 20-59*  *subscript “co” indicates from individuals aged 0-19 to individuals aged 60+*  *subscript “mo” indicates from individuals aged 20-59 to individuals aged 60+*  *Note: V compartments were stratified by vaccine products* |
