# Supplementary material for: Dynamics of the COVID-19 epidemic in the post-vaccination period in Korea: a rapid assessment
Source: Epidemiol Health. 2021 May 27;43:e2021040. doi: 10.4178/epih.e2021040 (PMC8289478; doi:10.4178/epih.e2021040)
Supplement: Supplementary Material 2. — Model calibration results and estimated effective contact rate for each age group. [file epih-43-e2021040-suppl2.docx]

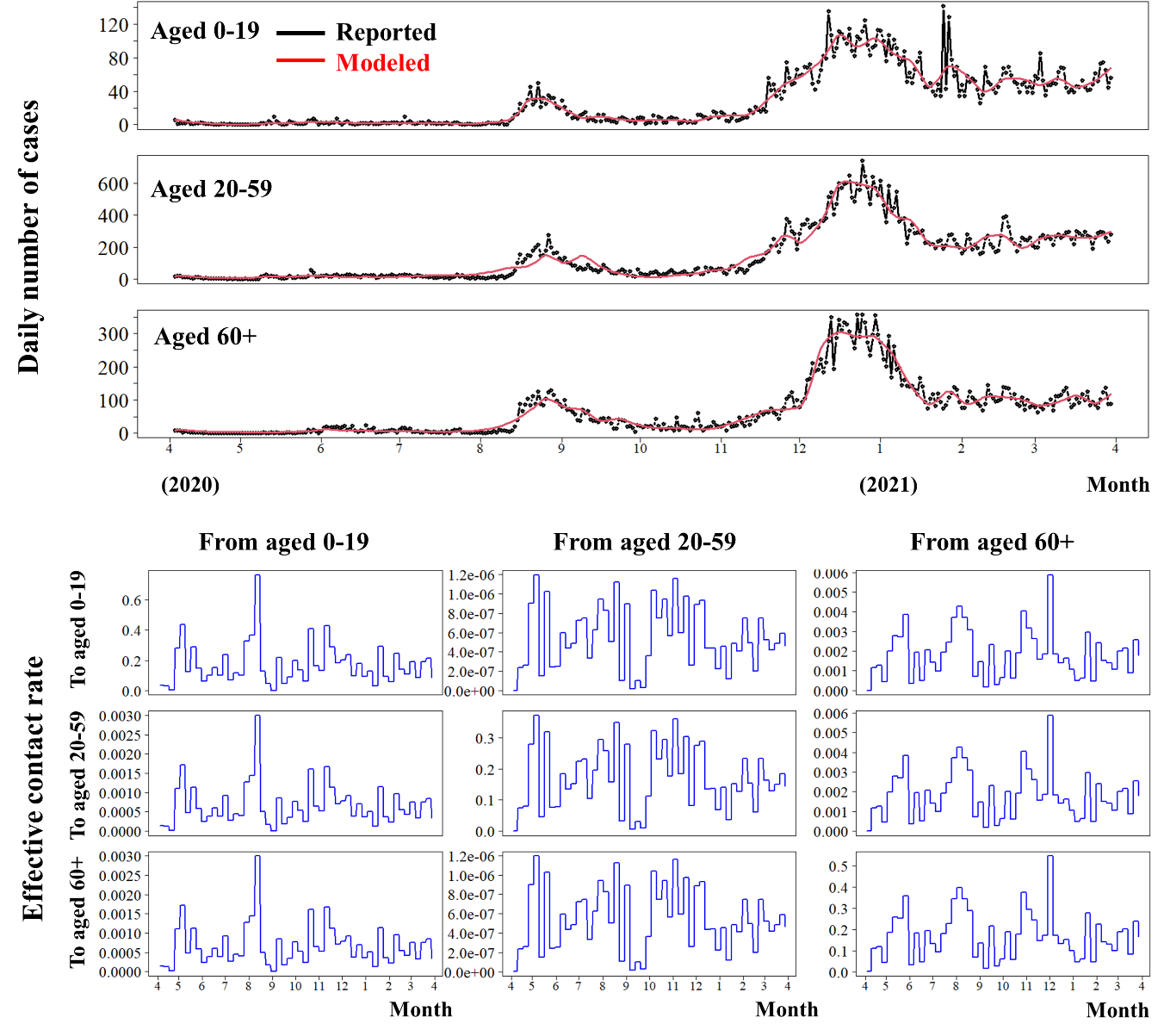


Supplementary Material 2. Model calibration results and estimated effective contact rate for each age group.
