# Supplementary material for: Dynamics of the COVID-19 epidemic in the post-vaccination period in Korea: a rapid assessment
Source: Epidemiol Health. 2021 May 27;43:e2021040. doi: 10.4178/epih.e2021040 (PMC8289478; doi:10.4178/epih.e2021040)
Supplement: Supplementary Material 3. — Effective contact rate for each age group and each period estimated by calibration [file epih-43-e2021040-suppl3.docx]

Supplementary Material 3. Effective contact rate for each age group and each period estimated by calibration

| Period | Social distancing level | | Estimated effective contact rate by age group | | | | Criteria for changing social distancing level^1^ |
| --- | --- | --- | --- | --- | --- | --- | --- |
|  | Capital  Area | Other  areas | 0–19 | 20–59 | 60+ | Mean |  |
| 05.06 (’20) ~ 08.18 | 1 | | 0.217 | 0.188 | 0.218 | 0.208 | 7.6 |
| 08.19 ~ 08.29 | 2 | | 0.075 | 0.148 | 0.147 | 0.123 | 122.4 |
| 08.30 ~ 09.13 | 2.5 | | 0.105 | 0.134 | 0.086 | 0.108 | 297.1 |
| 09.14 ~ 10.11 | 2 | | 0.117 | 0.126 | 0.117 | 0.120 | 125.0 |
| 10.12 ~ 11.18 | 1 | | 0.249 | 0.255 | 0.225 | 0.243 | 66.1 |
| 11.19 ~ 11.23 | 1.5 | 1 | 0.246 | 0.214 | 0.157 | 0.206 | 172.0 |
| 11.24 ~ 12.07 | 2 | 1/1.5 | 0.199 | 0.208 | 0.359 | 0.255 | 280.9 |
| 12.08 ~ 12.23 | 2.5 | 2 | 0.161 | 0.184 | 0.149 | 0.164 | 476.7 |
| 12.24 ~ 02.14 (’21) | 2.5+α^2^ | 2+α^2^ | 0.137 | 0.129 | 0.128 | 0.132 | 856.7 |
| 02.15 ~ 03.30 | 2+α^2^ | 1.5+α^2^ | 0.150 | 0.155 | 0.158 | 0.154 | 333.1 |

*Note*: A vaccinated–susceptible–latent–infectious–recovered (V-SLIR) model was employed in this study to simulate epidemics of coronavirus disease 2019 (COVID-19) in South Korea. Parameters such as latent period, infectious period, and effective contact rate were used in the model. While latent period and infectious period were fixed parameters based on previous literature, effective contact rate parameter was time-varying (we assumed different effective contact rates for each week) and was estimated by calibration to daily reported confirmed cases. By the calibration process, we obtained estimated effective contact rates for each week and each age group. Using the estimated effective contact rate, we represented mean contact rates for each age group in each time period categorized by implemented social distancing levels. Data fitting period was from April 3, 2020 to March 31, 2021. However, the estimated effective contact rates were shown after May 6, 2020, because social distancing intervention categorized by three levels was introduced after May 6, 2020.

^1^ mean number of daily confirmed cases between day of increasing/decreasing social distancing level and one-week before the day

^2^ prohibition of social gathering of five or more people
